# Zolpidem withdrawal seizure in an Iranian young woman: A case presentation

**DOI:** 10.22088/cjim.12.0.376

**Published:** 2021

**Authors:** Pezhman Hadinezhad, Seyed Hamzeh Hosseini

**Affiliations:** 1 Department of Psychiatry, Mazandaran University of Medical Sciences, Sari, Iran; 2 Psychiatry and Behavioral Sciences Research Center, Addiction Institute, Mazandaran University of Medical Sciences, Sari, Iran

**Keywords:** Zolpidem, Withdrawal, Seizure, Case report

## Abstract

**Background:**

Zolpidem is a non-benzodiazepine drug, approved by FDA for sleep induction. Zolpidem is thought to be a safer drug than benzodiazepines (BZD) because of no evidence of abuse or dependence potential, but several case reports of zolpidem abuse and dependence have been published along with a small number of cases demonstrating seizures after sudden zolpidem withdrawal.

**Case presentation:**

A 32-year-old unmarried woman suffering from major depressive disorder had been taking zolpidem for insomnia for more than 1 year. She began to take zolpidem alone without mixing other kinds of hypnotics, and 50 mg of zolpidem used to be initially effective in treating her insomnia. In some days the dose increased up to 100 mg per day. In the end, she had to discontinue zolpidem abruptly because she could not afford it anymore. After 2 days, she suddenly showed facial spasm, mouth opening, tonic-clonic seizure, and loss of consciousness for about 1-2 minutes. Post-ictal confusion with clouded consciousness, psycho-motor retardation, persisted in 1 day. EEG in wakefulness revealed intermittent, generalized, diffused alpha wave and diffused sharp waves, and suggested seizure waves in the patient.

**Conclusion:**

Our case suggested that the potential of zolpidem dependence and withdrawal seizure are also present in the Iranian population. The female-gender, high dosage and long-term use of zolpidem might be risk factors for the development of adverse effects.

Long-term use of benzodiazepines or benzodiazepine receptor agonists is widespread, although guidelines recommend short-term use. Only few controlled studies have characterized the effect of discontinuation of their chronic use on sleep and quality of life (1). Zolpidem is a non-benzodiazepine drug, approved by FDA for sleep induction (2) and a short acting hypnotic drug belonging to imidazopyridine family (3). It produces its hypnotic effects via the GABA-A benzodiazepine receptor complex, and binds preferentially to those receptors containing the alpha-1 subunit (4, 5). Zolpidem is thought to be a safer drug than benzodiazepines (BZD) because of no evidence of abuse or dependence potential (6). In comparison with benzodiazepines,this mechanism is thought to reduce liability to induce dependence (7). Earlier it was considered to be a safer hypnotic than benzodiazepines due to its lesser potentiality to cause abuse (8). But several case reports of zolpidem abuse and dependence have been published along with a small number of cases demonstrating seizures after sudden zolpidem withdrawal (9). Here we report a case of seizure related to withdrawal of zolpidem after long-term use in high dosage in Iran.

## Case presentation

A 32-years-old unmarried woman who had no history of any medical disorder or head trauma and no family history of epilepsy suffering from major depressive disorder had been taking zolpidem for insomnia for more than 1 year. Since one year ago after her brother’s sudden death, she began to take zolpidem alone without mixing other kinds of hypnotics, and 50 mg of zolpidem used to be initially effective in treating her insomnia. In some days, the dose increased up to 100 mg per day About 9 months later, she refers to a psychiatrist because of depression, anhedonia, fatigue, hopelessness and decreased appetite and the psychiatrist prescribed sertraline 50 mg once a day with diagnosis of major depressive disorder, the patient had taken the drugs with no problem for the past 3 months until than she was infected with herpes simplex virus and her doctor prescribed acyclovir 400 mg each 6 hours (1600 mg a day). In the end, she had to discontinue zolpidem abruptly because she could not afford it anymore. After 2 days, she suddenly showed facial spasm, mouth opening, tonic-clonic seizure, and loss of consciousness for about 1-2 minutes. Postictal confusion with clouded consciousness, psycho-motor retardation, persisted in 1 day. So she referred to her psychiatrist and a consultation with a neurologist had been requested by the psychiatrist. The neurological examinations were normal and EEG in wakefulness revealed intermittent, generalized, diffused alpha wave and diffused sharp waves, and suggested seizure waves in patient. As well as we use Naranjo scale for estimating the probability of relationship between seizure and zolpidem withdrawal, the patient score was 10, hence other etiologies or drugs either idiopathic causes were ruled out. After a series of laboratory tests and other examinations, no other etiologies could be identified in MRI. After 4 weeks follow-up, she consumed 5 mg zolpidem at bed time and she had no further seizure attacks and her postictal confusion resolved gradually 1 day after seizure occurred.

## Discussion

During the last decade, zolpidem (a non BZD hypnotic drug) was considered a new way for the treatment of patients with insomnia as it was suggested that it has the efficacy of BZDs for insomnia but without many side effects (10). It was suggested that zolpidem lacked muscle relaxant, anticonvulsant and anxiolytic properties and poor potential for abuse or dependence (11). GABA-A receptors include α1, α2, α3, α4, and α5 subunits receptors. The α1 subunit involves in sleeping mechanisms and α2 subunit contributes to anxiolytic action. BZDs have nonselective affinity to GABA-A subunits (12). A hypothesis about zolpidem withdrawal is long-term supra therapeutic doses saturation of the lower-affinity α2, α3 and α5 subunits on GABA-A receptors along with α1 subunits (13). Therefore, abrupt discontinuation of high doses would produce withdrawal symptoms such as anxiety, tremor, palpitation, or seizure (similar to BZDs withdrawal). Withdrawal symptoms of zolpidem were reported in less than 1% of subjects appearing within 48 hours of discontinuation (14). One of the probable factors associated with adverse effects of zolpidem is gender. Women have been found to have a significantly higher serum zolpidem concentration than men at equivalent dosage (15). 

Some studies showed that zolpidem withdrawal seizures occurred in higher doses than our case. In Haji Seyed Javadi et al.’s case presentation of a 30-year-old unmarried Iranian woman with dysthymic disorder and chronic insomnia was treated with zolpidem irregularly. She started to use zolpidem with 5mg per day irregularly since a year ago but augmented its daily dosage gradually to 100 to 150 mg per day in divided doses. After a period of 16 hours without taking zolpidem she developed a withdrawal syndrome, with generalized tonic-clonic seizures for two times. She was managed with supportive care and recovered completely (16). In our report, the patient consumed lower dose of zolpidem versus the current study. In other case presentations, Wang and et al. reported 2 cases of zolpidem withdrawal seizures and the result showed that potential of zolpidem dependence and withdrawal seizure are also present in the Asian population (17). According to these studies, gender is one of the susceptibility factors associated with adverse effects of zolpidem. Women had been found to have a significantly higher serum zolpidem concentration than men at equivalent dosage and in the end, ethnic differences have been demonstrated with the drug metabolizing enzymes, CYP2C9, 2C19, and 2D6 (18, 19). According to other case reports and studies and our case, zolpidem, soon after sudden discontinuation, causes withdrawal symptoms including insomnia, anxiety and epileptic attack, especially at high doses and long-term use. There have been several reports describing neuropsychiatric reactions such as visual hallucinations/sensory distortion, delirium, amnesia, sleepwalking/somnambulism, and nocturnal eating associated with zolpidem use (20). We suggest physicians to pay more attention to the potential of zolpidem to create dependence and withdrawal seizure. Besides, they should always keep its effects in their mind and subtilize during prescription of zolpidem for any patients and at any doses, especially for those with a previous history of drug or substance abuse and at high doses. However, this is a case study and it needs further studies to conclude about adverse effects of zolpidem.

Our case suggested that the potential of zolpidem dependence and withdrawal seizure are also present in the Iranian population. The female-gender, high dosage and long-term use of zolpidem might be risk factors for the development of adverse effects. It warrants further investigation whether there is ethnic variation for the liability of zolpidem dependence. Nevertheless, worldwide clinicians should pay attention to the risk of withdrawal seizure related to this agent, especially at high doses.
